# Mindfulness-based cognitive therapy versus psychoeducational intervention in bipolar outpatients with sub-threshold depressive symptoms: a randomized controlled trial

**DOI:** 10.1186/s12888-014-0215-x

**Published:** 2014-08-15

**Authors:** Guillermo Lahera, Carmen Bayón, Maria Fe Bravo-Ortiz, Beatriz Rodríguez-Vega, Sara Barbeito, Margarita Sáenz, Caridad Avedillo, Rosa Villanueva, Amaia Ugarte, Ana González-Pinto, Consuelo de Dios

**Affiliations:** Department of Medicine and Medical Specialties, University of Alcalá, Madrid, Spain; University Hospital La Paz, IDIPAZ, Madrid, Spain; CIBERSAM Biomedical Research Centre in Mental Health Net (CIBERSAM), University Hospital of Álava, Vitoria-Gasteiz, Spain

**Keywords:** Mindfulness, Psychoeducation, Bipolar disorder, Depression, Subsyndromal symptoms, Meditation, BDNF, Randomized clinical trial

## Abstract

**Background:**

The presence of depressive subsyndromal symptoms (SS) in bipolar disorder (BD) increases the risk of affective relapse and worsens social, cognitive functioning, and quality of life. Nonetheless, there are limited data on how to optimize the treatment of subthreshold depressive symptoms in BD. Mindfulness-Based Cognitive Therapy (MBCT) is a psychotherapeutic intervention that has been shown effective in unipolar depression. The assessment of its clinical effectiveness and its impact on biomarkers in bipolar disorder patients with subsyndromal depressive symptoms and psychopharmacological treatment is needed.

**Methods/design:**

A randomized, multicenter, prospective, versus active comparator, evaluator-blinded clinical trial is proposed. Patients with BD and subclinical or mild depressive symptoms will be randomly allocated to: 1) MBCT added to psychopharmacological treatment; 2) a brief structured group psychoeducational intervention added to psychopharmacological treatment; 3) standard clinical management, including psychopharmacological treatment. Assessments will be conducted at screening, baseline, post-intervention (8 weeks) and 4 month follow-up post-intervention. The aim is to compare MBCT intervention *versus* a brief structured group psychoeducation. Our hypothesis is that MBCT will be more effective in reducing the subsyndromal depressive symptoms and will improve cognitive performance to a higher degree than the psychoeducational treatment. It is also hypothesized that a significant increase of BDNF levels will be found after the MBCT intervention.

**Discussion:**

This is the first randomized controlled trial to evaluate the effects of MBCT compared to an active control group on depressive subthreshold depressive symptoms in patients with bipolar disorder.

**Trial registration:**

ClinicalTrials.gov: NCT02133170. Registered 04/30/2014.

## Background

Even in environments with standard psychiatric treatment and regular follow-up, around 38% of patients who have recovered from an acute episode of bipolar disorder (BD) show persistent subthreshold symptoms (SS) [1]. The Depression Comorbidity Study shows that the time with SS is higher than time in episode throughout the follow-up, with a clear predominance of depressive SS [2]. In a Spanish cohort, patients were symptomatic for one third of the 72-weeks follow-up, and also one third of the visits. In addition, they spent three times more days depressed than manic or hypomanic [3]. The persistence of SS has been associated with an increased risk of affective relapse/recurrence [4-6], a shorter time to relapse [7], greater functional impairment − similar to that experienced by patients with depressive episode−, worse quality of life, delayed functional recovery [8,9]) and poorer cognitive performance [10].

Nevertheless, few studies have assessed the efficacy of treatments on bipolar patients with depressive SS. Evidence of effectiveness using only drugs is limited, therefore alternatives are needed. Concomitant psychosocial interventions are recommended, such cognitive behavioral therapy (CBT), interpersonal and social rhythm therapy (IPSRT) and structured psychoeducation, especially for stabilization between episodes and relapse prevention [11]. In a recent study, a 21-session individual CBT was shown as effective -but much less cost-effective- as a brief psychoeducational group intervention, with overall decrease in disease burden, effectiveness in preventing episodes and time to relapse [12]. A combined treatment (pharmacological *plus* psychoeducational and cognitive-behavioral therapy) has shown to decrease depressive symptoms, even in refractory BD, and the number of hospitalizations [13]. Despite psychoeducational interventions appear to be most promising, the development of effective psychosocial therapies is still needed.

Mindfulness and acceptance-based interventions (MABIs) are receiving increasing attention in the treatment of mental disorders and show promise for symptoms of anxiety and depression both in clinical and nonclinical populations [14]. The practice of mindfulness works well in cognitive processes (rumination, cognitive reactivity, experiential avoidance), which have been associated with vulnerability to depressive and anxious symptoms and neuropsychological performance [15]. Mindfulness is currently defined in psychological terms as “paying total attention to the present moment with a non-judgmental awareness of inner and outer experiences” [16]. The primary mechanism of action is the moment-by-moment attention of our thoughts, feelings, bodily sensations, and surrounding environment, which promotes emotional regulation and awareness of the basic processes influencing the attribution of meaning to experiences. As important as the attention training is the promotion of an attitude of openness, curiosity and acceptance of all the experiences that arise. Mindfulness-Based Cognitive Therapy (MBCT), developed by Segal, Williams and Teasdale [17] is a group-based, 8-week, mind-body intervention that integrates psychological educational aspects of CBT for depression with meditation components of mindfulness-based stress reduction developed by Kabat-Zinn [16]). It has proven effective as adjunctive therapy in preventing relapse of recurrent depressive disorder [18-21] and reducing the residual depressive symptoms in unipolar depression [22,23]. In a pilot trial, MBCT compared to psychoeducation was significantly more effective in improving subsyndromal depressive symptoms in patients with unipolar depression [24]. In addition, MBCT is recommended in the NICE (National Institute for Health and Clinical Excellence) clinical guideline for the treatment of unipolar depressive patients with a history of 3 or more episodes [25].

Some preliminary studies and a randomized controlled trial with a long follow-up have examined MBCT in the treatment for bipolar disorder. Williams et al. [26] conducted a pilot clinical trial evaluating the effectiveness of MBCT versus waitlist on between-episode anxiety and depressive symptoms. Although the results were favorable for MBCT, these should be analyzed with caution given the small size and heterogeneity of the sample. Miklowitz et al. [27] studied the effect of a modified MBCT protocol with two additional treatment elements specifically designed for patients with bipolar disorder: psychoeducation about mood changes and its provoking factors (e.g. interpersonal conflict, sleep/wake cycle disruptions) and upon identification of these factors bringing mindfulness to prodromal signs of mood elevation. Small to medium effect sizes for improvement in depression and anxiety symptoms in remitted patients with bipolar disorder were found. In other preliminary study, at the end of a MBCT treatment, as well as at the 3-month follow-up, participants with BD showed lower residual depressive mood symptoms, less attentional difficulties, increased emotion-regulation abilities, psychological well-being, more positive affect, and psychosocial functioning and improvements in executive functioning, memory, and ability to initiate and complete tasks [28]. Other controlled studies have also shown a beneficial effect of MBCT for patients with BD, but with limitations such as small sample, absence of randomization or of an active comparator [29,30]. Perich et al. [31] have compared MBCT plus treatment as usual (TAU) to TAU alone in a RCT over a 12 month follow-up period. MBCT did not lead to significant reductions in time to depressive or hypo/manic relapse, total number of episodes, or mood symptom severity at 12-month follow-up; however, there was some evidence for an effect on anxiety symptoms. Despite decreases in depression were only observed at a trend level, further research is required to analyze the effect of MBCT on residual depressive symptoms in bipolar patients.

Mindfulness-based interventions are cost-effective, showing a potential to significantly reduce societal costs and increase the effectiveness of care [32]. Research in this field has shown important methodological limitations: differences in the application of the MBCT intervention, both in duration and number of sessions or the amount of incorporated psychoeducational elements; the limited statistical power due to small sample size; lack of control for possible confounding factors such as pharmacological treatment, or lack of information on the severity of the disease; and scarcity of long-term randomized clinical trials that allow to assess the maintenance of achieved effects. In the present project we try to overcome several of these limitations, especially by using the method of randomized clinical trial with an active comparator, the larger sample size and the attempt to control confounding variables regarding the condition and design of the intervention.

Structured group psychoeducation is an adjunctive psychological treatment that has been shown to be effective in the prevention of affective recurrences as well as number of hospitalizations and time with symptoms in BD euthymic outpatients, both at two-year [33] and five-year follow-up [34]. This psychoeducational program is structured in 21 sessions delivered through 6 months. It stresses the importance of illness awareness, self-management, early-warning sign identification, habit regularity, treatment adherence and avoiding drug misuse. “It has been defined as “behavioural psychoeducation”, but its authors rather think of it as an “attitudes & aptitudes” psychoeducational program. This definitely corresponded to a view of bipolar disorder as a complex condition involving not only biological etiological factors but also psychological and social variables that may act as triggering factors, modulators or mediators” [35]. This study has been successfully replicated in pharmacologically treated patients with bipolar disorder also in routine clinical settings [36].

The brain -derived neurotrophic factor (BDNF) is a dimeric protein that promotes the growth and maintenance of neuronal connections, modulates neurotransmission and participates in the mechanisms of learning and long-term plasticity (LTP). Peripheral BDNF in serum and plasma can be assessed noninvasively by venipuncture. Since it crosses the blood–brain barrier, its levels in serum and plasma are highly correlated with levels in cerebrospinal fluid (r ¼ 0.8) [37,38]. There are numerous studies assessing peripheral levels of BDNF in bipolar disorder patients. Some studies suggest that BDNF levels decrease during mood acute states and remain normal during euthymia, but other studies have contradicted this paradigm. A recent meta-analysis has measured the effect sizes of the differences in BDNF levels between BD patients in different mood states and controls. The BDNF levels were decreased in both mania and depression when compared to controls [39,40] but were not different in euthymia [41]. Meta-regression analyses in euthymia showed that age and length of illness influenced the variation in effect sizes [42]). BDNF levels are consistently reduced during manic and depressive episodes and recover after treatment for acute mania, so peripheral BDNF could be used as a biomarker of mood states and disease progression for BD.

In summary, the presence of persistent depressive subthreshold symptoms increases the risk of affective relapse and negatively affects the prognosis of BD. However, there is no an evidence-based specific treatment for them. MBCT appears as a promising intervention, since it has been found to be effective in reducing current depressive symptoms in MDD [23,43]. Our objective is to evaluate the efficacy of the MBCT added to standard drug treatment -following the recommendations of the Spanish Guideline for the Treatment of BD [11])- *versus* structured group psychoeducation versus standard clinical management. Our primary outcome is reduction of residual depressive symptoms. Secondary outcomes are manic and anxiety symptoms, recurrence, cognitive and social functioning and levels of BDNF.

## Methods/design

This is a parallel 3-group, multicenter, prospective, randomized; single-blind (evaluator) controlled pilot trial, with a 4 month follow-up post-intervention. Patients diagnosed with bipolar disorder (BD) according to DSM −5 criteria for mild depression or subsyndromal depressive symptoms are assigned to one of the following 3 treatment groups: 1) psychopharmacological treatment *plus* Mindfulness Based Cognitive Therapy (MBCT); 2) psychopharmacological treatment *plus* a brief, structured group psychoeducation; 3) treatment as usual (TAU), defined as standard psychiatric care with standard pharmacologic treatment. After written informed consent is signed, patients meeting the inclusion criteria are randomized (2:2:1 ratio) through a Random Allocation Software. All three groups are assessed at baseline (t0), immediately after completing the program (t1; 8 weeks) and at follow-up six months after randomization. The assessments include the following variables: depression, manic and anxiety symptoms, general and social cognition, global functioning, BDNF, and other clinical variables. The evaluator who will collect the biomarkers and the clinical and psychometric data will be blind to allocation of treatment. The interrater variability between all researchers will be checked.

### Subjects

One hundred and forty bipolar out-patients with persistent mild/subsyndromal depressive symptoms will be recruited from Mental Health Centers as well as Private Psychiatric Clinics and Bipolar Patient Associations via announcements or physician referral. The study will be conducted in La Paz University Hospital (Madrid) and the Santiago Apóstol University Hospital (Vitoria).

### Inclusion criteria

1-Age: 18–60 years; 2 – BD type I or II, in clinical remission of acute mood episode at least in the three months prior to study; 3 - Having experienced an acute affective episode in the past 3 years, 4 - Having suffered at least two lifetime depressive episodes. 5- Monotherapy or combination with a mood stabilizer (lithium, valproate, carbamazepine or lamotrigine) at optimal doses (i.e., in serum levels within the therapeutic range: 0.6-1.2 mEq/L for lithium, 50–100 ug/mL for valproate, and 5–12 mcg/mL for carbamazepine), or quetiapine monotherapy or in combination with the aforementioned stabilizers, or any oral atypical antipsychotic in combination with an antidepressant; 6.- Hamilton Depression Rating Scale [HDRS]–17 [44] score ≥ 8 and ≤ 19 and Young Mania Rating Scale [YMRS; 49] score <8; 7 - Being able to understand and comply with the requirements of the trial, 8 - Written consent to participate in the study.

### Exclusion criteria

1. Any acute mood episode in the 12 weeks before the start of the trial. 2. Any current DSM −5 diagnosis different from bipolar disorder (including substance or alcohol use disorder at the time of study entry, except if it is under complete remission. Not applicable to nicotine or caffeine). 3. Risk of suicide or self/hetero aggressiveness. 4. Pregnancy. 5. Severe and unstable medical disease. 6. Patients who are currently receiving structured psychotherapy or structured group psychoeducation about bipolar disorder, or who have received structured psychoeducation in the past 5 years; 6. Patients who are treated with a different mood stabilizer than lithium, valproate, carbamazepine, lamotrigine, a classic antipsychotic or antidepressant monotherapy at the time of the randomization. 7. Treatment with a depot antipsychotic; 8. Participation in another clinical trial within 4 weeks prior to randomization. 9. Mental retardation.

### Withdrawal criteria

Express wish of the participant, unjustified absence from more than two group training sessions, appearance of suicidal ideation or psychotic symptoms, onset of acute manic or hypomanic episode, or need for psychiatric hospitalization from any cause.

### Primary outcome measures

Primary endpoint of the study is given by changes in the overall score of the Hamilton Rating Scale for Depression (HDRS [44]), from baseline (V0) to week 8 (v1) for each of the treatment groups.

### Secondary endpoints

1. Changes in the global score of Young Mania Rating Scale (YMRS, [45]) from baseline (V0) to visit 1 (at the end of the 8 week intervention (v1)
2. Changes in the score of Clinical Global Impression CGI-BP [46] from baseline (V0) to visit 1;
3. Changes in the score of the Hamilton Rating Scale for Anxiety HAM-A [47] from baseline (V0) to visit 1;
4. Cognitive changes: changes at the end of the intervention will be assessed with the following measures:
  sustained and selective attention
  working memory and executive functions
  perception of the attitude of mindfulness (Five Facet Mindfulness Questionnaire FFMQ, [48]) in patients in the experimental group
  scales of social cognition
5. Functioning: changes in total scale score of the Functioning Assessment Short Test (FAST; [49]).
6. The following clinical variables are also assessed:
  Recurrence, defined as the emergence of a new acute episode whether depressive, mixed, hypo or manic at any time throughout the study, according to DSM-5 clinical criteria or when the score on the HDRS scale is ≥ 20 (depressive episode) or the Young scale ( YMRS ≥ 8) (hypo/manic episode), or a change drug or hospitalization is needed.
  Plasma levels of BDNF: changes from baseline to visit 1 (end of intervention).

Twenty-four weeks after the start of intervention following measurements are assessed: changes in the overall score of the Hamilton Depression Rating Scale (HDRS [44]), Young Mania Rating Scale (YMRS, [45]) from baseline (V0 ) to week 24 (V2) , Hamilton anxiety Scale HAM- A [47], CGI [46], general cognitive functioning, social cognition, functioning and biomarkers (BDNF).

### Intervention

MBCT program will be conducted in HULP by two experienced therapists with recognized expertise in Mindfulness based stress reduction (MBSR), MBCT and narrative psychotherapy. Both therapists will train two therapists from Vitoria with expertise in cognitive therapy and MBSR. The MBCT program consists of 8 weekly sessions of 90 minutes and will be performed in groups of approximately 10–15 patients. All therapy sessions will be audio recorded to be discussed and analyzed by the treatment team, in order to standardize the intervention. Treatment fidelity will be also assessed using the Mindfulness- based Cognitive Therapy Adherence Scale (MBCT-AS; [50]), which assesses the key constructs of MBCT during group sessions.

Psychoeducation program will be held in groups of 10 to 15 patients in 90-minute weekly sessions led by two therapists blind to the clinical and cognitive evaluation. The specific program of 8 sessions addresses disease awareness, adherence to treatment and early detection of prodromal symptoms. Homework will also be included. The program is based on the Psychoeducation Manual for Bipolar Disorder [51]. Attendance to at least 80% of the sessions of both interventions will be required to be considered complete.

### Assessment instruments

#### a) Clinical variables

1 Depressive symptoms: measured by the Hamilton Depression Rating Scale of 17 items (HDRS-17) [44,52]. 3. Manic symptoms: measured by the Young Mania Rating Scale (YMRS) [45]. 4. Hamilton Anxiety Scale HAM-A [47]. 5. Clinical Global Impression Scale modified for bipolar disorder (CGI-BP) [46]. 6. Adherence to treatment: Good (according to information from the patient, family and blood drug levels), Bad (if none of the above criteria are met) or Average (only if it meets one or two criteria). 7. Clinical outcome: new mood episodes, serious adverse events (including suicidal ideation, suicide and hospitalization).

#### b) Cognitive variables

##### General cognition

- Sustained attention (Continuous Performance Test, [53]). The test provides the following measures: correct responses, errors of omission (not responding when the stimulus appears) and commission errors (responding without the letter stimulus appearance).
- Selective attention (Stroop Test, [48]). Requires the subject to be able to suppress irrelevant response. When the name of a color (e.g., “blue,” “green,” or “red”) is printed in a color not denoted by the name (e.g., the word “red” printed in blue ink instead of red ink), naming the color of the word takes longer and is more prone to errors than when the color of the ink matches the name of the color [54].
- Working memory. 1. Subtest from the Weschler Adult Intelligence Scale [55]. A sequence from 2 to 8 digits or a list of words have to be repeated in reverse or random order of presentation.
- Executive functions: 1. Wisconsin Card Sorting Task [56]. Participants have to sort the cards according to different criteria that vary throughout the test (perseverative errors and completed categories are the dependent variables).

#### c) Social cognition

The recognition of emotions through the face is measured with the Face Emotion Identification Task and Face Emotion Discrimination Task (FEIT and FEDT respectively, [57]). The FET scale includes 19 photographs of faces expressing one of the six basic emotions (happy, sad, angry, scared, shocked or embarrassed). The task is to identify the patient’s emotion expressed in each. The FEDT includes 30 pairs of faces and the subject must determine if both sides show the same or different emotion.

#### d) Mindfulness

Five Factors Questionnaire Mindfulness (FFMQ, [58]) 39 items assessing five mindfulness facets: observing, describing, acting with awareness, equanimity and no reactivity. Each item is scored on a Likert scale ranging from 1 (never) to 5 (very often). A high internal consistency and high predictive validity have been shown [58] and it has been uvalidated in Spanish [59].

#### e) Functioning

Measured with the Functioning Assessment Short Test (FAST, [49]), which measures the degree of difficulty found by the patient in the following areas: autonomy, occupational functioning, cognitive functioning, finances, relationships and leisure.

#### f) Biomarkers: serum analysis of BDNF

### Data analysis

An exploratory study of all collected variables identifies possible outliers and errors. The descriptive analyses of qualitative variables are expressed as frequencies and percentages and quantitative variables as mean, standard deviation, median and interquartile range. Analysis is performed by intention to treat. Comparison of qualitative variables between the intervention groups or between other factors of segmentation is by means of Chi 2 or Fisher ‘s exact test. For quantitative variables, the T-test, Mann -Whitney or Kruskal –Wallis and ANOVA are applied, depending on the normality of the variable (tested by Kolmogorov -Smirnov - Lilliefords). The change analyses along the track of the primary and secondary outcome variables are performed using a General Linear Model (ANOVA with repeated measures). Regression models are extracted to identify prognostic factors for the efficacy variables. The statistical package SPSS 18.0 is used in all tests and a bilateral level of p < 0.05 is considered statistically significant.

### Ethical issues

The study is conducted according to local regulations and internationally established principles at the the Helsinki Statement (64° Asamblea General Fortaleza, Brasil, 2013), whilst respecting confidentiality (Data Protection Act 15/99). Protocol was approved by the Clinical Research Ethics Committee of the University Hospital La Paz (Madrid) and the Clinical Research Ethics Committee of the Basque Country.

## Discussion

This study will provide feasibility information and preliminary effect size estimates to inform the effectiveness of adjunctive MBCT, adjunctive group psychoeducation and standard treatment on SS or mild depressive symptoms in BD. It is of interest to researchers involved in the development of new approaches to the treatment of residual affective symptoms and its negative impact in the course of illness. It can also provide information on variations of BDNF in the different treatment arms and on neuropsychological measures, which might suggest mechanisms of action and differences among treatments.

Strengths of the study include targeting a defined sample of bipolar patients with persistent depressive symptoms despite receiving a correct pharmacological treatment. This sample –as reported before- is representative of a high percentage of bipolar out-patients, who remain symptomatic more than one third of the time. The trial protocol was registered before randomization began (ClinicalTrials.gov: NCT02133170 Registered 04/30/2014) and was developed according to good clinical research practice enabling the randomized trial to be conducted with low risk of bias and a high degree of external validity. Both MBCT and psychoeducational interventions will be conducted using manuals and adherence to the treatment manuals will be tested. This makes it possible to implement the interventions in clinical practice, and the two interventions can be assessed in future trials. To standardize the application of MBCT and psychoeducation, specific training and meetings will be held. Specific training also may increase the inter-rater reliability on scales.

Since an 8-week psychoeducational program based in the Barcelona group-structured model has not proved effective neither in depressive symptoms nor in episode prevention, this could be a study weakness. But our objective is to compare MBCT versus an active psychosocial intervention, and this is the best model we could find. Sample size could also be a limitation, as well as the time of follow-up, especially regarding the possible effects on long-term course of BD.

## Abbreviations

ANOVA: Analysis of variance
BD: Bipolar disorder
BDNF: Brain derived neurotrophic factor
CBT: Cognitive behavioral therapy
CGI-BP: Clinical global impression scale modified for bipolar disorder
FAST: Functioning assessment short test
FEDT: Face emotion discrimination task
FEIT: Face emotion identification task
FFMQ: Five factors questionnaire mindfulness
HAM-A: Hamilton anxiety scale HAM-A
HDRS: Hamilton depression rating scale
IPSRT: Interpersonal and social rhythm Therapy
ABIs: Mindfulness and acceptance-based interventions
MBCT: Mindfulness based cognitive therapy
SS: Subsyndromal symptoms
YMRS: Young mania rating scale
